# Response of two temperate scleractinian corals to projected ocean warming and marine heatwaves

**DOI:** 10.1098/rsos.231683

**Published:** 2024-03-27

**Authors:** Chloe Carbonne, Steeve Comeau, Keyla Plichon, Sébastien Schaub, Jean-Pierre Gattuso, Núria Teixidó

**Affiliations:** ^1^ CNRS, Laboratoire d’Océanographie de Villefranche, Sorbonne Université, 181 chemin du Lazaret, Villefranche-sur-mer, Monaco 06230, France; ^2^ MSc MARRES, Université Côte d’Azur, Sophia Antipolis Campus, Nice 06103, France; ^3^ CNRS, Laboratoire de Biologie du Développement de Villefranche, Sorbonne Université, 181 chemin du Lazaret, Villefranche-sur-mer, Monaco 06230, France; ^4^ Institute for Sustainable Development and International Relations, Sciences Po, 27 rue Saint Guillaume, Paris 75007, France; ^5^ Department of Integrated Marine Ecology, Stazione Zoologica Anton Dohrn, Ischia Marine Centre, Punta San Pietro, Ischia, Naples 80077, Italy

**Keywords:** ocean warming, marine heatwaves, Mediterranean Sea, coral, bleaching

## Abstract

The Mediterranean Sea is a hotspot of global change, particularly exposed to ocean warming and the increasing occurrence of marine heatwaves (MHWs). However, experiments based on long-term temperature data from the field are scarce. Here, we investigate the response of the zooxanthellate coral *Cladocora caespitosa* and the azooxanthellate coral *Astroides calycularis* to future warming and MHWs based on 8 years of *in situ* data. Corals were maintained in the laboratory for five months under four temperature conditions: Warming (3.2°C above the *in situ* mean from 2012 to 2020), Heatwave (temperatures of 2018 with two heatwaves), Ambient (*in situ* mean) and Cool (deeper water temperatures). Under the Warming treatment, some *C. caespitosa* colonies severely bleached and *A. calycularis* colonies presented necrosis. *Cladocora caespitosa* symbiosis was impaired by temperature with a decrease in the density of endosymbiotic algae and an increase in per cent whiteness in all the treatments except for the coolest. Recovery for both species was observed through different mechanisms such as regrowth of polyps of *A. calycularis* and recovery of pigmentation for *C. caespitosa*. These results suggest that *A. calycularis* and *C. caespitosa* may be resilient to heat stress and can recover from physiological stresses caused by heatwaves in the laboratory.

## 1. Introduction

Climate change severely impacts marine biodiversity and ecosystem functioning globally [1]. The upper layers of the ocean store over 90% of the excess heat caused by global warming, resulting in a global warming trend of sea surface temperature (SST) of 0.15°C per decade over the past 40 years [2]. In addition to gradual ocean warming, the frequency and severity of extreme climatic events such as marine heatwaves (MHWs) have increased considerably (e.g. [1]). MHWs are defined as seawater temperatures exceeding a threshold, usually the 90th percentile of the climatological mean, for at least five consecutive days [3]. Three properties characterize MHWs: (i) their duration, measured as the consecutive days of the event, (ii) the maximum intensity, defined as the highest temperature anomaly in °C, and (iii) the cumulative intensity, which is the sum of the daily intensities during the event in °C days [3]. MHWs have significant impacts on species and ecosystems, driving major ecological and physiological changes (e.g. [1,4,5]). These extreme events have a large range of effects on physiological processes that can result in mortality and local extinctions if the upper thermal threshold and acclimatization potential of a species are exceeded (e.g. [6,7]). MHWs have already affected many ecosystems and are responsible for the loss of kelp forests (e.g. [4]), coral bleaching (e.g. [6,8]) and widespread mortality of habitat-forming macrophytes and invertebrates in the Mediterranean Sea (e.g. [5,9]).

The Mediterranean Sea is considered a hotspot of climate change where important ecological effects on marine biodiversity are projected [10,11]. The increase in SST is projected to be 20% higher in the Mediterranean Sea than in the global ocean [12]. SST is projected to increase globally by 3.2°C by the end of this century under the RCP8.5 emission scenario [13]. From 1993 to 2017, SST increased by 0.53°C per decade in the Mediterranean Sea while global SST has increased by 0.14 ± 0.01°C per decade (ERSST V5 NOAA dataset [14]). During the period 2015–2019, exceptional thermal conditions and MHWs in the Mediterranean Sea resulted in five consecutive years of widespread mass mortality events of marine species [5]. Recently, in 2022, western parts of the Mediterranean Sea have experienced the second hottest summer since the beginning of measurements, the hottest year being 2003 [15]. In 2022, the anomaly of SST between 1.3 and 2.6°C above the long-term average (1982–2011), and temperatures up to 30.8°C were recorded in August in the northwestern Mediterranean Sea [15]. These extreme thermal events cause a long-term decline in habitat-forming species including corals and gorgonians that are among the most affected benthic organisms by MHWs [5]. It is, therefore, necessary to understand the physiological response of Mediterranean corals to gradual warming as well as MHWs and to assess their capacity to recover from such conditions.

Heat stress on zooxanthellate corals is responsible for bleaching (e.g. [6,8]), which is the breakdown of symbiosis between the coral host and its endosymbiotic algae [16]. Most carbon requirements of corals are obtained from photosynthetic products translocated from their symbionts (e.g. [17,18]). Thus, bleaching leads to a drastic reduction of metabolic resources, loss of reserves, decrease in growth rate, shifts in the microbiome and higher disease susceptibility, which, combined, can cause an increase in mortality (e.g. [19,20]). Most of the knowledge accumulated on this issue has been gathered on symbiotic tropical corals, while the response to MHWs of temperate Mediterranean zooxanthellate and azooxanthellate corals (e.g. [21–24]) is much less studied.

The present study focuses on two Mediterranean habitat-forming corals of key relevance for conservation, the zooxanthellate species *Cladocora caespitosa* and the azooxanthellate species *Astroides calycularis. Cladocora caespitosa* is one of the few zooxanthellate corals of the Mediterranean Sea and the only one forming localized bioconstructions comparable to coral reefs in the tropics [25,26]. Its sensitivity to MHWs has been assessed across the Mediterranean during field studies, and some laboratory experiments have investigated its thermal tolerance (e.g. [21,27–29]). In the laboratory, elevated temperature during summer led to an increase in growth rate, calcification, respiration, photosynthesis and asexual reproduction [30,31]. A thermal threshold of 24°C, above which photosynthesis is inhibited, was determined in laboratory [27]. However, this temperature is below the current summer mean temperature. *In situ* studies have confirmed that elevated summer temperature could lead to direct necrosis (loss of living tissue) in the Ligurian Sea, Balearic Islands and Columbretes Islands [21,32,33] or to bleaching in Croatia, Montenegro and Cyprus [22,28,34]. However, both bleaching and necrosis could also appear in the same site, picturing a very complex reaction of *C. caespitosa* to heat stress between the Mediterranean populations (electronic supplementary material, table S1). *Astroides calycularis* is commonly found in low light, rocky habitats, from surface to 50 m depths [35]. Its main geographic distribution is the southwestern basin of the Mediterranean Sea [35]. This coral is considered a warm-water species with a narrow temperature tolerance confined to a minimum of 14°C during the winter [36]. In the laboratory, *A. calycularis* showed a decrease in calcification by 25% and an increase in the porosity of the skeleton when exposed to an increase of 3°C compared with the natural annual temperature cycle measured at the sampling site [23]. Mortality events of *A. calycularis* during late summer have been reported during the last years on the coast of Ischia and Pelagie Islands [5,24] with the warm summer of 2020 causing a widespread mortality event.

This study, based on 8 years of *in situ* temperature data, was designed to test three hypotheses: (i) the physiology of both corals is affected by future warming and heatwaves, (ii) the symbiotic species *C. caespitosa* is more susceptible to elevated temperature, and (iii) the two corals can recover during autumn from summer heat stress. We studied, in the laboratory for five months, the physiological performance and recovery of these two habitat-forming species under present and future summer temperatures. We contrasted the response of respiration, calcification and relative number of polyps between the zooxanthellate and the azooxanthellate species. Net photosynthesis, whitening of the colonies and density of symbionts were measured in *C. caespitosa* to evaluate the role of symbiosis under heat stress.

## 2. Material and methods

### 2.1. Sampling site

Forty colonies of *A. calycularis* (whole colonies of approx. 5 cm in diameter) and one fraction of 40 colonies of *C. caespitosa* (approx. 5 cm in diameter) were collected at 10 m depth on 1 July 2020 by scuba diving in Ischia, Italy. The sites were: Sant’Angelo for *A. calycularis* (latitude 40.691972 and longitude 13.893056) and Chiane del Lume for *C. caespitosa* (latitude 40.717444 and longitude 13.967278). Colonies were maintained in a 30 l tank filled with ambient seawater with water motion provided by a NEWA mini 606 pump for 24 h before transportation to the aquarium facilities at the *Laboratoire d’Océanographie de Villefranche*, France. Colonies were cleaned, their bases covered with water-resistant epoxy, tagged and maintained at an ambient temperature of 23°C for two weeks for acclimatization before the experiment. For *C. caespitosa*, eight additional reference samples were taken on 1 July and 18 September 2020 (hereafter called ‘*in situ*’ samples) and stored at −80°C for further endosymbiotic algae density measurements.

### 2.2. Seawater temperature in Ischia

Seawater temperature at the two collection sites was recorded hourly from June 2012 to June 2021 at 10 and 15 m depths (electronic supplementary material, figures S1 and S2) with HOBO Water Temperature Pro v. 2 Data Loggers (Onset). The daily mean temperature was calculated for each day of the year with the temperature data over the period 2012–2020, for 10 and 15 m depth. The number of MHWs and cumulative intensities were analysed with the HeatwaveR package [37]. MHW events, periods of at least 5 days over the 90th percentile, and their metrics such as cumulative intensity were determined with the detect_event function applied to daily data of the climatology period of Ischia created with the ts2clm function with temperature data from 2012 to 2020 (electronic supplementary material, figure S3).

### 2.3. Experimental set-up and treatments

**Figure 1.**
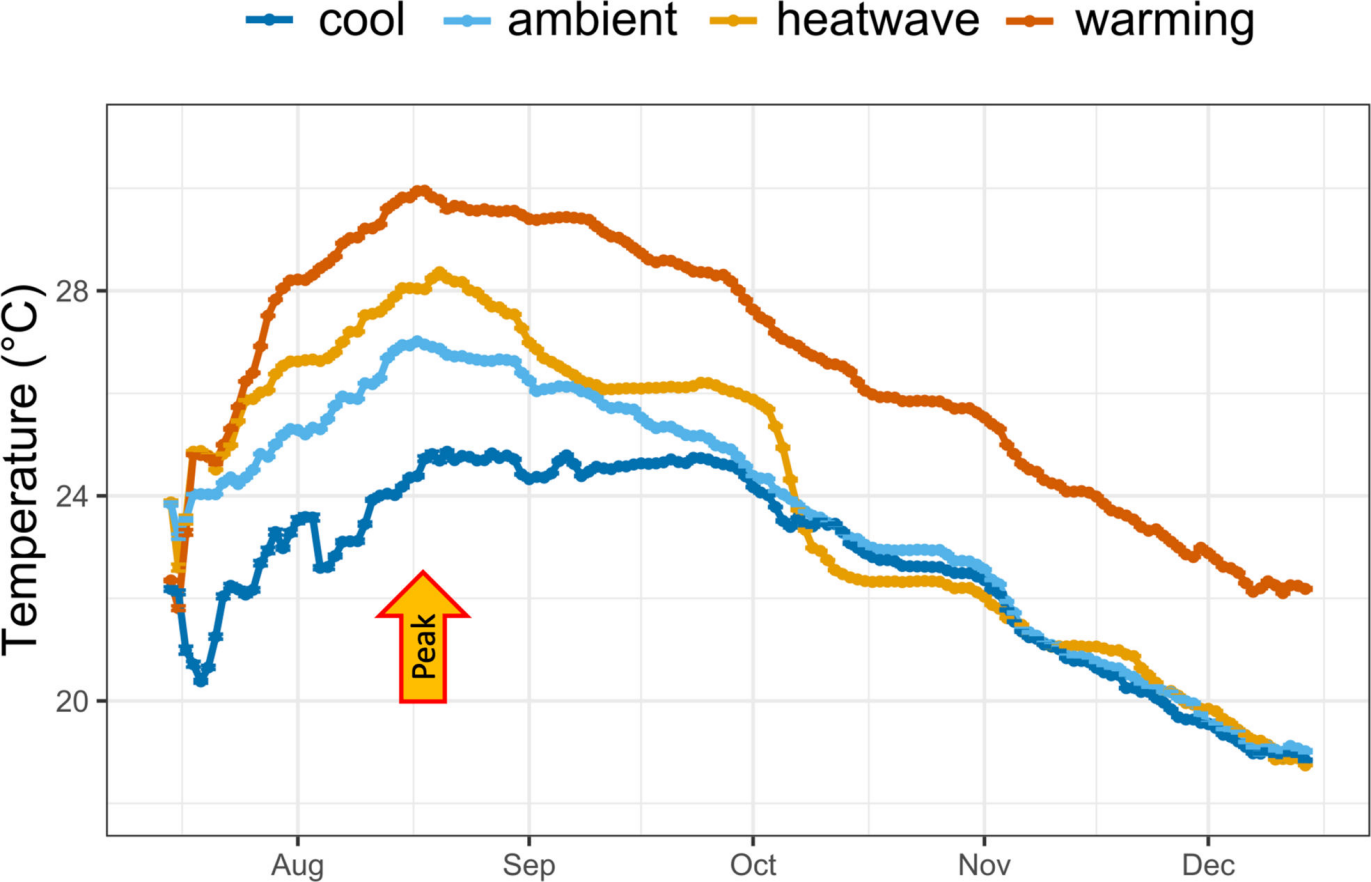
Temperature treatments applied during the five-month experiment. ‘Ambient’ is the daily mean temperature from 2012 to 2020 at 10 m depth, ‘Warming’ is the mean temperature at 10 m depth + 3.2°C, ‘Heatwave’ is the temperature of the year 2018 presenting the highest number of heatwaves recorded from 2012 to 2020 and ‘Cool’ is the daily mean temperature from 2012 to 2020 at 15 m depth. The ‘peak’ arrow indicates the peak of temperature of the experiment (16 August 2020).

Colonies of *A. calycularis* and fragments of *C. caespitosa* were maintained in four temperature treatments for 146 days (figure 1). The ‘Ambient’ treatment mimicked the daily mean temperature over 2012–2020 at 10 m depth in Ischia. The ‘Warming’ treatment corresponded to the ambient treatment +3.2°C, the mean increase in global SST projected at the end of the century under the RCP 8.5 scenario [13]. The ‘Heatwave’ treatment mimicked the daily mean temperature of 2018, which was the year presenting the highest number of MHWs at 10 and 15 m depths (electronic supplementary material, figure S2). Finally, the ‘Cool’ treatment replicated the daily mean temperature over 2012–2020 at 15 m depth. Eight colonies or fragments of each species were randomly distributed in separate 5 l experimental tanks for each of the four temperature treatments, for a total of 32 experimental tanks. Seawater pumped from Villefranche Bay at 5 m depth was continuously flowing into eight 25 l header tanks that fed four experimental tanks at a rate of 100 ml min^−1^. The experimental tanks were placed in water baths where temperature was controlled using temperature controllers (APEX, Neptune Systems) which controlled 300 W heaters (Thermocontrol, Eheim). With this system, the temperature difference was less than 0.1°C between tanks of the same treatment. The temperature was adjusted daily, to mimic the natural daily change in temperature. Light was provided by 89 W LED light bars (Aquaristik, Aqualumix). Irradiance gradually increased from 0 at 06.50 to a maximum irradiance of 150 µmol photons m^−2^ s^−1^ at 12.30 and gradually decreased to 0 at 19.00. Irradiance was measured with a LI-1400 datalogger mounted with an underwater quantum sensor LI-192 UWQ8268 (LI-COR). The maximum irradiance levels used in the study (150 μmol photons m^−2^ s^−1^) is within the irradiance range observed at 10 m, where *C. caespitosa* can be found in the Mediterranean Sea (attenuation coefficients of photosynthetically available radiation (PAR) from Martin and Gattuso [38]. Dark plastic bags covered one side of each experimental tank to shade colonies of *A. calycularis* from direct light. MINI 404 submersible pumps (420 l h^−1^, NEWA) provided water motion in each experimental tank. Twice a week, one scoop of Marine Power coral food LPS pellets (Tropical) was added to each tank for feeding, and polyps of *A. calycularis* were hand-fed once a week with small pieces of fish filet. Two colonies of each species died on 20 November 2020 owing to technical issues in the aquarium system.

### 2.4. Changes in polyp numbers and calcification

Pictures of each side of each individual maintained in seawater were taken every month in a glass aquarium with a Coolpix W300 camera (Nikon), during the five-month-long experiment. These pictures were used to count the number of polyps per colony. The relative number of polyps per colony ( ρ ), which is a way to evaluate the growth of a coral colony, was defined as


$$\rho \left(m\right)=\frac{N\left(m\right)}{N\left(m-1\right)}-1,$$

where *m* is the month and *N* is the number of polyps, including new buds and death of polyps by necrosis. In all temperature treatments, we observed a decrease in symbiont density from the start of the experiment to one month after peak temperature. This was also observed *in situ*. ρ was measured on the periods between the beginning (T0 = 27 July 2020), the peak of temperature (peak = 16 August 2020) and the two following months (T1 = 17 September 2020 and T2 = 12 October 2020)

Calcification rates during the three first months of the experiment (between T0 and T2, before sampling polyps from each colony) were assessed using the buoyant weight technique [39]. The weight change was normalized by the surface of living tissue and the number of days (88 days). The surface area was determined using the aluminium foil technique [40].

### 2.5. Physiological measurements

Dark respiration of both species and net photosynthesis of the zooxanthellate *C. caespitosa* were measured by incubating each colony separately in 500 ml transparent perspex chambers at the beginning of the experiment (29 July 2020), at the peak temperature (16 August 2020) and about one month after the peak temperature (30 September 2020), when temperature was the same as during the initial incubations. The chambers were placed in a temperature-controlled water bath to maintain the corals at temperatures similar to the ones in the experimental tanks. Incubations lasted 30–60 min depending on the metabolic activity and the size of the samples. Colonies were acclimatized in the dark for 10 min prior to the start of dark respiration measurements. Net photosynthesis was measured on *C. caespitosa* under an irradiance of 150 μmol photons m^−2^ s^−1^. Magnetic stirrers provided water motion during the incubation. An incubation with no coral was used as a blank for each temperature treatment. Oxygen saturation within the chambers was measured every 5 s using a fibre optic oxygen sensor (PreSens, OXY-4 mini), calibrated at 0% and 100% saturation. The difference in oxygen saturation between the beginning and the end of the incubation was corrected with the blank and then converted to O_2_ mg l^−1^ using the gas_O2sat function of the marelac R package [41]. Oxygen consumption was then normalized by the coral surface area, incubation time and chamber volume to obtain dark respiration and net photosynthesis. Gross photosynthesis was calculated assuming respiration is the same in light and dark.

### 2.6. *Cladocora caespitosa* bleaching and symbionts density

**Figure 2.**
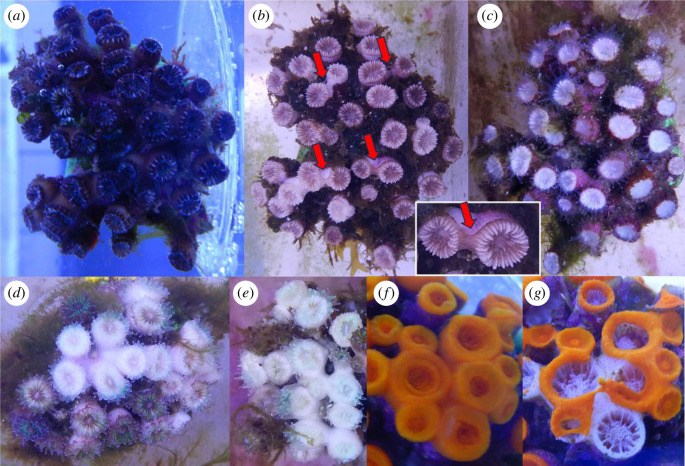
Pictures of *C. caespitosa* necrosis and bleaching and *A. calycularis* necrosis. (*a*) Picture of a healthy colony of *C. caespitosa* under the warming treatment at the beginning of the experiment (28 July 2020), (*b*) bleached and presenting coenosarc necrosis two weeks after the peak of temperature (31 August 2020) and (*c*) severely bleached two months after the peak (12 October 2020) presenting transparent tentacles. (*d*) and (*e*) Severely bleached polyps of *C. caespitosa* from the Heatwave treatment with no sign of necrosis. (*f*) Colony of *A. calycularis* under the Warming treatment at the beginning of the experiment (28 July 2020) and (*g*) two months after the peak (9 October 2020) showing important polyp necrosis.

Quantification of *C. caespitosa* bleaching was performed as described by McLachlan & Grottoli [42] by measuring means of greyscale on the ImageJ software (v. 1.53) using the monthly photographs of the organisms (see §2.4, figure 2*a–c*). The per cent of whiteness, equivalent to bleaching in greyscale pictures, was shown to be negatively correlated with its zooxanthellae density and chlorophyll concentration [43]. The measurement of whiteness provides a proxy of bleaching by a non-invasive method. The entire surface of every polyp of each colony facing the camera was manually selected on greyscale pictures, and a mean greyscale was obtained for each polyp (from 0, black, to 255, white). A measure of per cent whiteness was obtained for each colony by computing the mean of greyscales of all of its polyps and corrected with a white standard.

The symbiont density for *C. caespitosa* was measured on one polyp of each of the eight reference colonies sampled *in situ* and from one polyp removed with clamps on each of the experimental colonies after three months (12 October 2020) and at the end of the experiment (15 December 2020). Tissues were removed with an air-brush and homogenized with an Ultra Turrax (IKA) in 0.5 ml of MilliQ water. Samples were then centrifuged at 800*g* for 2 min (Centrifuge 5417C, Eppendorf^®^). The supernatant was removed, and the pellet was the symbiont material that was stored at −80°C in 1 ml of MilliQ water. Four 200 µl replicates of each sample were disposed in a haemocytometer (Malassez cell, Marienfield), and images were collected on an inverted ZEISS Observer microscope (Zeiss, Jena, Germany) equipped with a 10×/0.3 EC Plan-Neofluar objective lens using a monochrome Flash 4 (Hamamatsu photonics, Japan). Chlorophyll fluorescence was acquired with 405 nm excitation and 659–701 nm emission. Mosaics of 9 mm² were typically acquired for brightfield and chlorophyll fluorescence using motorized stage driven under the Zen software. We developed a set of macros under ImageJ to easily settle the parameters and analyse all the images automatically in batches, providing a datasheet for statistics with, among other measures, the absolute volumetric concentration of endosymbiotic algae. The macros are available on GitHub with tutorial and example: https://github.com/SebastienSchaub/CounZoox/ (and electronic supplementary material, method S1, for more information). The endosymbiotic algae density was normalized by the volume of the sample and the polyp photosynthetic surface (PS). The PS was determined as described by Rodolfo-Metalpa *et al*. [27].

### 2.7. Data analysis

Linear mixed models with a hierarchical structure were used to evaluate the temperature treatment effects through time on the relative number of polyps per colony, the rates of respiration and net and gross photosynthesis as well as the per cent whiteness. Hierarchical linear models (HLMs) were used since data were compiled from repeated measures of the same pool of colonies over time. The models were fitted using the lmer function of the R package lme4 [44]. The fixed factors of the models were temperature and time, and the experimental tank and header tank were assigned as random effects. The structure of the random term was selected by comparing models with different error structures using the Akaike information criterion (electronic supplementary material, table S2). Tukey’s *post hoc* tests were conducted when significant differences were detected.

Permutational multivariate analysis of variance (PERMANOVA) with the adonis2 function of the vegan R package was used to test for the effects of the treatments on calcification rates and the endosymbiotic algae density because these parameters are not time-dependent or based on repetitive measurements of the same organisms. Temperature and time were used as fixed factors.

## 3. Results

### 3.1. Seawater temperature in Ischia

Over 8 years of temperature data collection at Ischia, the monthly mean maximum occurred in August at 10 m depth with a temperature of 26.3°C and in September at 15 m depth at 24.1°C (electronic supplementary material, figure S1). The monthly mean minimum was 14.6 and 14.5°C in March, for 10 and 15 m, respectively. The maximum temperature was 28.3°C at 10 m in the summer of 2018 and 27°C at 15 m in the summer of 2015. From 2013 to 2020, mean summer temperatures increased by 0.032°C per year at 10 m depth and 0.051°C per year at 15 m (electronic supplementary material, figure S2). Summer 2018 was the summer presenting the highest number of MHWs (two at 10 m and three at 15 m) and cumulative intensities (the sum of the daily intensities during the event, greater than 40°C × days at 10 m and greater than 60°C × days at 15°C, electronic supplementary material, figure S3).

### 3.2. Change in the number of polyps and calcification

**Figure 3.**
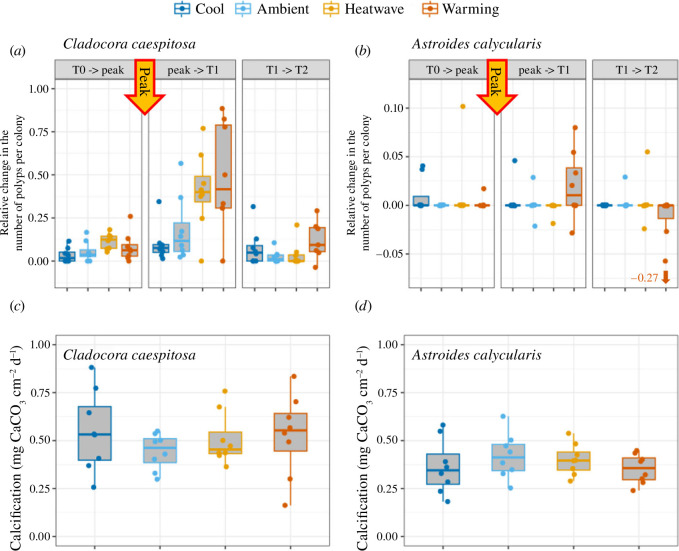
Growth of *A. calycularis* and *C. caespitosa* colonies exposed for three months to the four temperature treatments. Panels (*a*) and (*b*) show the monthly relative change in the number of polyps per colony from the beginning of the experiment to the peak of temperature (from T_0_ to peak), during the first month after the peak (from peak to T1) and during the second month after the peak (from T1 to T2), panels (*c*) and (*d*) show the calcification rates normalized by surface area for the first three months of the experiment. Dots represent the relative change in the number of polyps and calcification rates of each colony per experimental tank; boxes represent the median with the 25% and 75% quartiles. The colour of the box indicates the temperature treatment. *n* = 8 per treatment. Note: −0.27 (outlier data point in panel *b*) refers to a value we did not include in the graph to help reading. T0 = 27 July 2020; peak temperature = 16 August 2020; T1 = 17 September 2020; T2 = 12 October 2020.

Colonies of *C. caespitosa* under the Warming (mean temperature at 10 m depth +3.2°C) and the Heatwave (temperature of the year 2018) treatments exhibited an increase in the number of polyps by budding up to six times higher during the month following the peak temperature (from the peak to T1) than before the peak (from T0 to the peak) and two months after (from T1 to T2, figure 3*a*). The number of polyps budding under the Warming and the Heatwave treatments during the month following the peak temperature was three times higher than under the Ambient (daily mean temperature from 2012 to 2019 at 10 m depth) and Cool (daily mean temperature from 2012 to 2019 at 15 m depth) temperature treatments. Temperature and time significantly affected the relative number of polyps per colony (*F*_9,110_ = 41.22, *p* < 0.001, figure 3*a*, electronic supplementary material, table S3) as colonies presented more new polyps or buds after the peak when maintained under higher temperatures (Cool < Ambient < Heatwave < Warming). Of the *C. caespitosa* colonies from the Warming treatment, 37.5% presented necrosis of coenosarc (3/8 colonies, figure 2*a*,*b*), and only one colony presented loss of polyps (6/18 polyps) during the second month after the peak (from T1 to T2).

*Astroides calycularis* presented very little to no change in the number of polyps per colony (−0.265 to 0.125, mean = 1.79 × 10^−3^, *n* = 126), with 85% of colonies showing no change), and no statistical effects of treatment or time were found (*F*_9,108_ = 14.10, *p* = 0.12, figure 3*b*, electronic supplementary material, table S3). However, colonies of *A. calycularis* under the Warming treatment a month after the peak temperature (from the peak to T1) presented very variable responses, with new polyps budding in some colonies while others had lost polyps and showed denuded skeleton owing to necrosis (figure 2*f*,*g*). In the second month after the temperature peak (from T1 to T2), four colonies of *A. calycularis* exhibited necrosis in the Warming treatment (ranging from 1 to 14 polyps lost).

The calcification rate of both species was not significantly affected by temperature (PERMANOVA, *F*_3,28_ = 0.42, *p* = 0.74 for *C. caespitosa* and *F*_3,28_ = 1.36, *p* = 0.27 for *A. calycularis*, figure 3*c*,*d*, electronic supplementary material, table S4).

### 3.3. Respiration and photosynthesis

**Figure 4.**
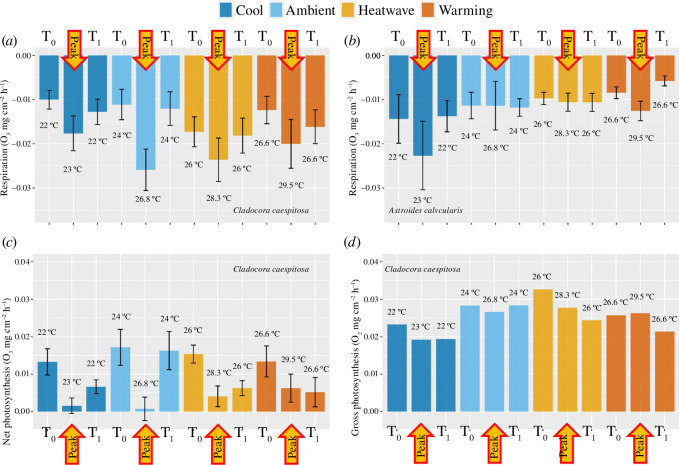
Net and gross photosynthesis and dark respiration rates of *C. caespitosa* and *A. calycularis* under the four temperature treatments at the beginning of the experiment (T_0_), peak of temperature and one month after the peak (T_1_). Values are mean ± s.e. Panel (*a*) shows dark respiration of *C. caespitosa*, panel (*b*) shows dark respiration of *A. calycularis* and panels (*c*) and (*d*) show net and gross photosynthesis of *C. caespitosa*. The colour of the bars indicates the origin and the temperature treatment. *n* = 8 per treatment. T0 = 27 July 2020; peak temperature = 16 August 2020, T1 = 17 September 2020.

The respiration rate of *C. caespitosa* was significantly affected by the interaction of temperature and time (*F*_9,110_ = 17.51 *p* < 0.001, figure 4*a*). The respiration rate at the peak temperature was 72% higher than one month before (T0) and 47% higher than after the peak (T1) for all temperature treatments combined (T0: −0.011 ± 0.002, peak: −0.022 ± 0.002, T1: −0.015 ± 0.001 mg O_2_ cm^−1^ h^−1^, mean ± s.e.). In all periods combined (T0, peak and T1), corals in the Cool treatment presented lower respiration rates than in the other treatments (−0.013 for Cool, −0.016 for Ambient and Warming and −0.020 mg O_2_ cm^−1^ h^−1^ for Heatwave). Net photosynthesis was also significantly affected by the interaction of temperature and time (HLM, *F_6,82_* = 15.02 *p* = 0.02, figure 4*c*), with photosynthesis lower at the peak temperature than one month before (T0) or after the peak (T1) for all the treatments. However, gross photosynthesis was not impacted by temperature nor time, nor by their interaction (figure 4*d*, electronic supplementary material, table S3).

Concerning *A. calycularis*, respiration was only affected by time (*F*_2,82_ = 7.24, *p* = 0.03). For the Cool and Warming treatments, respiration was almost 50% higher during the peak temperature (T0: −0.014 ± 0.005, peak: −0.023 ± 0.007, T1: −0.014 ± 0.004 mg O_2_ cm^−1^ h^−1^, mean ± s.e.); however, for the Ambient and Heatwave treatments, no differences were found between the three time points (figure 4*b*, electronic supplementary material, table S3).

### 3.4. *Cladocora caespitosa* bleaching and symbionts density

**Figure 5.**
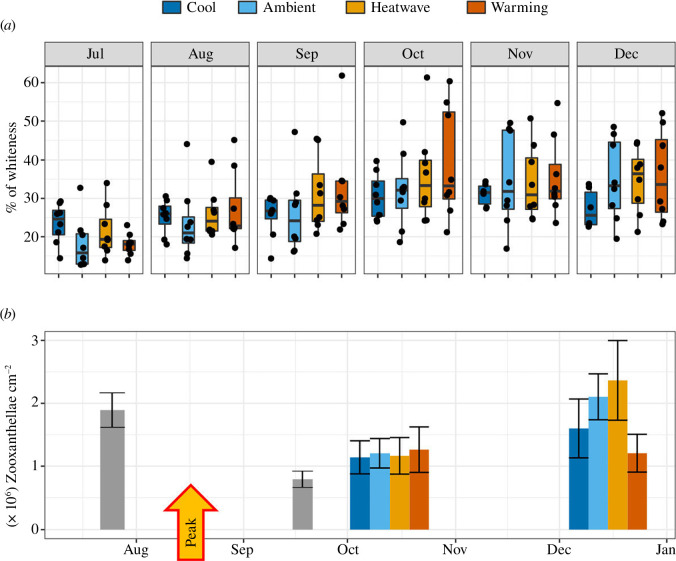
Response of the endosymbiotic algae of *C. caespitosa* over time under the four temperature treatments. Panel (*a*) shows the per cent of whiteness of the colonies over time. Dots represent the per cent of whiteness of each colony and boxes represent the median with the 25% and 75% quartiles. Panel (*b*) shows the density of endosymbiont algae per PS. The grey bars indicate the ‘*in situ*’ samples collected in the field. Values are mean ± s.e. The colour of the box indicates the treatment. *n* = 8 per treatment. Peak temperature = 16 August 2020.

A whitening (equivalent to bleaching in greyscale pictures) of the colonies was observed in all the treatments except for the Cool treatment. The percentage of colony’s whiteness was significantly impacted by the temperature treatment and time (*F*_3,1325_ = 33.08, *p* < 0.001, figure 5*a*, electronic supplementary material, table S3). The mean whiteness of the colonies at the beginning of the experiment was around 20%. The maximum whitening was reached three months after the peak temperature, when it increased to 40% in the Warming treatment, 37% in the Heatwave treatment and 35% in the Ambient treatment. The most severely impacted colonies (three in Warming and one in Heatwaves) reached up to 60% of whiteness in the Warming treatment three months after the peak temperature (figure 2*c*). There was no significant difference in the per cent whiteness in the Cool treatment.

The symbiont density also significantly changed with the interaction of treatments and time (*F*_4,70_ = 3.11, *p* = 0.02, figure 5*b*, electronic supplementary material, table S4). The endosymbiotic algae density was around 2 × 10^6^ cells cm^–2^ at the beginning of the experiment. It decreased to 1 × 10^6^ cells cm^–2^ under the four temperature treatments in the middle of the experiment (start of October) as well as for the *in situ* reference samples collected at the end of September. Interestingly, corals from all temperature treatments were able to regain their initial symbiont density (2 × 10^6^ cells cm^–2^) at the end of the experiment except in the Warming treatment, where it remained at about 1 × 10^6^ cells cm^–2^.

## 4. Discussion

For five months, colonies of the zooxanthellate coral *C. caespitosa* and the azooxanthellate coral *A. calycularis* were exposed to four different treatments to assess their physiological response to gradual warming and MHW as well as their recovery. Our approach to applying the daily means of temperature based on 9 years of field data with different warming scenarios and extreme events, such as MHWs, was applied for the first time to Mediterranean corals. It allowed assessing a realistic response of the two species to projected future temperatures and their recovery after summer peak temperature. The use of the natural cycle of temperature data based on 9 years of *in situ* records is important to better understand the effects of extreme events that are increasing every year in frequency and intensity in the Mediterranean Sea. For example, recently, summer 2022 might have been one of the worst MHWs, with SST anomaly of +5°C along the coast of France and Italy [15]. Our results showed an impact of high temperature on the number of polyps and respiration for both species and on the symbiosis of *C. caespitosa*. Interestingly, both species recovered rapidly with respiration rates returning to pre-peak levels, regrowth of polyps of *A. calycularis* and regain of pigmentation after bleaching for *C. caespitosa* after three months. Combined, these results suggest the resilience of both zooxanthellate and azooxanthellate species by specific responses to the summer peak temperatures under laboratory conditions.

### 4.1. Warming increases the metabolism

High temperatures are known to favour the increase in budding of *C. caespitosa* [30,45]. This was observed here for both *C. caespitosa* and *A. calycularis* under the warming treatment. However, *A. calycularis* presented a much lower increase in the number of polyps than *C. caespitosa*, possibly owing to their different trophic strategy. *A. calycularis* is an azooxanthellate species that relies solely on heterotrophy for nutrient provision. However, in summer, owing to the establishment of a thermocline and overall low hydrodynamics, particulate organic matter is severely reduced in the Mediterranean Sea [46]. Even if corals were fed three times a week during the experiment, summer is probably a low growth period for *A. calycularis*. Moreover, the reproduction of *A. calycularis* occurs from April to the end of June [47], and it is an energy-consuming process. Therefore, no energy may have been available for other functions such as the growth of new polyps in summer. In contrast, summer is a period of active growth for *C. caespitosa* with the maximum increase in calix height occurring between July and September [32]. On the other hand, summer is also the gametogenesis period for *C. caespitosa*, which is a highly energy-demanding process [48]. The difference in budding between the two species could also be owing to a difference in morphology. *Astroides calycularis* has bigger polyps (6.3 ± 1.3 mm in diameter, *n* = 83) than *C. caespitosa* (5.3 ± 0.7 mm in diameter, *n* = 81), so budding for this first species would be a higher energy-consuming procedure.

*Cladocora caespitosa* also showed an increase in respiration with an increase in temperature. Our results are in agreement with an increase in respiration of *C. caespitosa* between cold temperatures in winter and higher temperatures during summer [31] or during a heat stress experiment at 26 and 28°C [27]. Even if temperature affected the number of polyps per colony of both species and the respiration rates for *C. caespitosa*, no impact was found on the calcification rate for the two species. This could be owing to a reallocation of calcium carbonate from the thick skeleton of older polyps [49] to the newly budded polyps on *C. caespitosa* colonies during the peak temperature. The calcification rate was similar to the calcification rate previously observed in these species [23,27,30]. However, in previous studies, both species showed a decrease in the growth rate when temperatures were raised from 24°C to 28°C [23,27].

### 4.2. Are azooxanthellate corals more resistant to warming and marine heatwaves than zooxanthellate corals?

As a proxy of warming, a latitudinal gradient of temperature along the coast of Italy where zooxanthellate and azooxanthellate corals are found (8° range) has been studied [50]. Azooxanthellate corals were more resistant to increased temperature, with *Leptopsammia pruvoti* and *Caryophyllia inornata* having the same growth rate [51,52] and the same reproductive efficiency at the different sites [53,54]. In contrast, zooxanthellate corals were more affected by increased temperature with inhibition of calcification and a decrease in reproduction efficiency along the gradient of temperature for *Balanophyllia europaea*, possibly owing to inhibition of photosynthesis at elevated temperature [55,56]. In this present study, the zooxanthellate *C. caespitosa* presented increased respiration and whitening under Ambient, Heatwave and Warming treatments. In contrast, the azooxanthellate *A. calycularis* only responded to the Warming treatment with an increase in the number of polyps during the peak temperature and necrosis in four colonies after the peak. *Astroides calycularis* also presented a higher tolerance of respiration to temperature than *C. caespitosa*. This difference in tolerance between species might be owing to the fact that the photosynthetic efficiency and density of *C. caespitosa*’s symbionts are reduced above 24°C [27], possibly owing to photosynthesis inhibition of symbiotic algae at high temperatures. Thus, *A. calycularis,* which is not dependent on symbionts for food input, has a higher temperature threshold than *C. caespitosa*. All the more so, *A. calycularis* considered a thermophilic species, has been observed migrating towards the north of the Mediterranean Sea with ocean warming [36].

### 4.3. Population of *C. caespitosa* from Ischia

Depending on the site in the Mediterranean Sea*, C. caespitosa* has been reported to bleach (loss of endosymbiotic algae in Croatia [22], in Montenegro [34]) or to present direct necrosis with the loss of tissue when exposed to heat stress (in Italy [57] and in Spain [21,33]) or both (in Cyprus [28]). Our study confirms the bleaching of *C. caespitosa* (figure 2*c–e*). Here, the loss of tissue by necrosis only happened after bleaching (figure 2*b*). Furthermore, some severely bleached polyps never showed any sign of necrosis or polyp retraction as observed in Rodolfo-Metalpa *et al*. [27]. The microbiome of the coral may play a role in these different responses to increasing temperature. Necrosed colonies of *C. caespitosa* and *Oculina patagonica* are associated with a decrease in Pseudovibrio species compared with healthy colonies [58]. Furthermore, under warming seawater, the inoculation of *Vibrio* AK-1, thermodependent bacteria in *O. patagonica* led to bleaching after 10 days, while no bleaching occurred when *Vibrio* was absent [59]. Assessing changes in the microbiome of *C. caespitosa* across locations during MHW may shed light to better understand the cause of these geographical discrepancies. In the present study, colonies of *C. caespitosa* were generally less impacted by warming than reported by Rodolfo-Metalpa *et al*. [27], where all colonies died when exposed to 26 and 28°C for 48 days. The mortality observed by Rodolfo-Metalpa *et al*. [27] might be owing to the difference in experimental design or to the origin of the colonies. The corals from Rodolfo-Metalpa *et al*. [27] were sampled in the North of Italy (Gulf of La Specia and Genoa) and thus from cooler temperatures, while we worked on colonies from Ischia experiencing regularly warm temperatures in summer. The difference in temperature from one site to another has a great impact on the background of a population, as observed by Kersting *et al*. [29]; between 2010 and 2013, the population of *C. caespitosa* in Columbretes Island at 15 m depth suffered up to 27.7°C and showed necrosis events every year, while the Medes population, with a maximum of 24.8°C remained healthy. The per cent whiteness (used as a proxy for bleaching) monitored in this study was associated with the density of endosymbiotic algae. In all temperature treatments, we observed a decrease in symbiont density from the start of the experiment to one month after peak temperature. This was also observed *in situ*. Aside from the Warming treatment that did not recover in autumn, the other treatments’ colonies followed a natural seasonal variation for temperate corals, with a decrease in symbiont during summer to the beginning of autumn and recovery in winter. This event is mostly controlled by changes in irradiance and temperature [30]. The same phenomenon is also observed in tropical environments, where the highest densities of symbionts usually occur during the winter [60].

### 4.4. Photosynthesis of endosymbionts and bleaching

The first sign of the impact of temperature on the symbiosis of *C. caespitosa* was a decrease in the photosynthetic efficiency (electronic supplementary material, figure S3). Elevated temperatures are known to induce saturation of photosynthetic electron transport leading to the degradation of the photosystem II and promoting the generation of reactive oxygen species that trigger bleaching by losing symbiont or by photopigment degradation [61,62]. Maximum quantum yield in the Warming, Heatwave and Ambient treatments only decreased after peaks at 30, 28.3 and 26.8°C, respectively. Under the Cool treatment, it remained constant under temperatures never above 24°C. This is consistent with the fact that a temperature threshold of 24°C was reported in *C. caespitosa* [27] and that no significant diminution of photosynthesis yield was observed before the summer peak of temperature in a study also mimicking summer heat on *C. caespitosa* [29]. Interestingly, gross photosynthesis did not change with time nor across temperature treatments from the beginning to two months after the peak of summer temperature. Under summer temperatures with a peak around 25°C, Rodolfo-Metalpa *et al*. [31] observed an increase in net photosynthesis on *C. caespitosa* while a decrease was measured by Jurriaans *et al*. [63]. In this study, as most of the symbiotic responses (decrease in the endosymbiotic algae density and increase in the per cent whiteness) occurred after the period of the first three months, it might explain why gross photosynthesis was not impacted yet. As expected with the decreasing values of quantum yield right after the peak, bleaching occurred one month after peak temperature in the Ambient and Heatwaves treatments and severe bleaching in the Warming treatment.

### 4.5. Mechanisms of recovery after summer heat

For both species, a quick recovery of respiration after the peak temperature was observed. In all treatments, respiration rates were similar one month before and one month after the peak, even if respiration increased during the peak. The same response was observed in *C. caespitosa* during a recovery phase of 14 days at 24°C after heat stress at 26°C [27]. With a heat stress of 28°C, respiration of *C. caespitosa* was not back to initial rates after recovery [27]. The fact that recovery was higher in our study compared with the experiment of Rodolpho-Metalpa *et al*. [27] might be owing to our recovery time being twice as long, which is important for long-lived species, as the two corals of this study.

*Cladocora caespitosa* also recovered from heat stress. After three months post-peak, colonies from Ambient and Cool treatments had returned to the same density in the endosymbiotic algae as at the beginning of the experiment and showed a decrease in the percentage of whiteness, thus, a full recovery of symbiont population health after bleaching. The severely bleached colonies from the warming treatment recovered little pigmentation (electronic supplementary material, figure S4), and symbiont density was not back to the initial levels after four months. This is in agreement with the results of Rodolfo-Metalpa *et al*. [27] who showed that after 14 days of the recovery phase at 24°C, corals exposed to heat stresses of 26 and 28°C, had their density of symbiont almost five times lower than at the beginning of the experiment. The few necrosed areas observed on *C. caespitosa* did not recover any tissue, and the exposed skeleton was eventually covered in algae.

After losing tissue by necrosis (electronic supplementary material, figure S4), *A. calycularis* presented a regeneration of fully functional polyps from the growth of remaining live tissue in dead calyx areas (electronic supplementary material, figure S5). Previous observations show that if the mouth of polyps of three solitary fungiid species is necrosed, a new mouth can develop from the remaining tissue present in the calyx of the necrosed polyp [64]. A similar process was also observed in *C. caespitosa* when, after a MHW, polyp tissues retracted inside the calyx as a transitory resistance phase, before regrowing. This mechanism may facilitate faster recovery of colonies severely affected by warming [65]. Other regenerative mechanisms have been observed owing to heat stress on both studied species such as polyp bail-out or propagules [66,67]. This process consists of a full polyp detaching from its skeleton forming a single skeleton-less polyp [67] and allowing preserving tissue to regenerate a full colony after stress. These regenerative mechanisms are important for the recovery and population survivorships after MHWs and an opportunity for corals to deal with global warming.

## 5. Conclusion

This laboratory study highlights a moderate impact of MHWs on both *C. caespitosa* and *A. calycularis* with the rapid recovery of the physiological parameters impacted by MHW. In the Warming treatment, however, three colonies of *C. caespitosa* showed partial necrosis and loss of six polyps while four colonies of *A. calycularis* had lost up to 14 polyps. Necrosis observed *in situ* for both species exposed to MHWs up to 29°C [21,24] has been more important than described in this study, with a recurrent increase in mass mortalities these past few years [5]. The projected warming of +3.2°C for the end of the century tested in this study had the strongest impact, with severe bleaching of *C. caespitosa* and higher necrosis of *A. calycularis* leading only to a partial recovery after four months. Owing to the expected increasing number and intensity of future warming events [1], bleached coral would need a longer recovery time between the events because of the reduced capacity for coral to recover from repetitive stress (e.g. [20,68]). Ocean warming is projected to increase along with other stressors such as ocean acidification [13] or food availability. During summer, in the Mediterranean Sea, other stressors such as a decrease in particulate organic matter owing to stratification [46] or coverage of the coral colonies by invasive filamentous macroalgae [29] are combined with warming. Feeding the corals in this study could have mitigated the impact of heat stress compared with real *in situ* conditions [30]. Moreover, the combined effect of ocean warming and acidification decreases recruitment and post-settlement growth of early life stages of *A. calycularis* [69]. However, warming seems to be the main factor leading to the decrease in calcification and increase in respiration of adult colonies of *C. caespitosa* and *A. calycularis* [23,31]. On the other hand, the recovery of a coral community or coral reef is not only dependent on the heat stress degree but also on long-term biotic and abiotic conditions following the stress such as coral recruitment and survival, grazing of algal competitors, nutrient availability and sedimentation [70]. Understanding how future temperatures will impact these two Mediterranean habitat-forming species and how they recover is essential in order to be able to preserve them as well as coastal benthic ecosystems.

## Data Availability

Raw data and R script have been published on Dryad [71]. Electronic supplementary material is available online [72].
